# Metastasis organotropism in colorectal cancer: advancing toward innovative therapies

**DOI:** 10.1186/s12967-023-04460-5

**Published:** 2023-09-09

**Authors:** Kai He, Zhihan Wang, Maochao Luo, Bowen Li, Ning Ding, Lei Li, Bo He, Han Wang, Jiangjun Cao, Canhua Huang, Jun Yang, Hai-Ning Chen

**Affiliations:** 1 School of Basic Medical Sciences and State Key Laboratory of Southwestern Chinese Medicine Resources, Chengdu University of Traditional Chinese Medicine, Chengdu, 611137 China; 2 State Key Laboratory of Biotherapy and Cancer Center, West China Hospital, and West China School of Basic Medical Sciences and Forensic Medicine, Sichuan University, and Collaborative Innovation Center for Biotherapy, Chengdu, 610041 China; 3 Department of Oncology, The First Affiliated Hospital of Kunming Medical University, Kunming, 650032 China; 4 Department of General Surgery, State Key Laboratory of Biotherapy and Cancer Center, Colorectal Cancer Center, West China Hospital, Sichuan University, Chengdu, 610041 People’s Republic of China

**Keywords:** CRC, Organotropism, Metabolic reprogramming, Immune system, Cancer cell-organ interactions, Targeted therapy

## Abstract

Distant metastasis remains a leading cause of mortality among patients with colorectal cancer (CRC). Organotropism, referring to the propensity of metastasis to target specific organs, is a well-documented phenomenon in CRC, with the liver, lungs, and peritoneum being preferred sites. Prior to establishing premetastatic niches within host organs, CRC cells secrete substances that promote metastatic organotropism. Given the pivotal role of organotropism in CRC metastasis, a comprehensive understanding of its molecular underpinnings is crucial for biomarker-based diagnosis, innovative treatment development, and ultimately, improved patient outcomes. In this review, we focus on metabolic reprogramming, tumor-derived exosomes, the immune system, and cancer cell-organ interactions to outline the molecular mechanisms of CRC organotropic metastasis. Furthermore, we consider the prospect of targeting metastatic organotropism for CRC therapy.

## Introduction

**Fig. 1:**
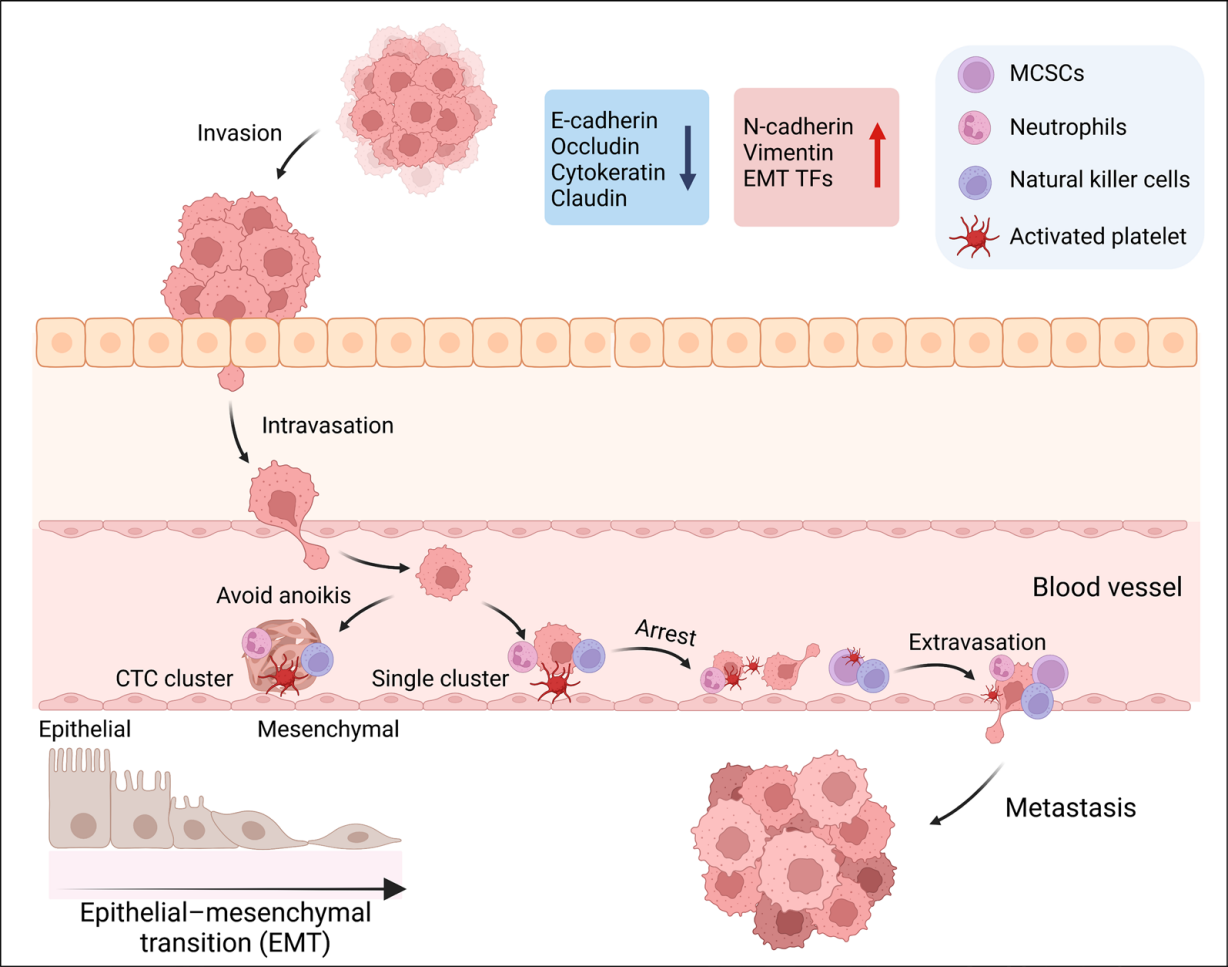
A schematic representation highlights the role of EMT in tumor metastasis. EMT initiates the metastatic cascade by inducing in situ tumor cells to undergo phenotype transformation. In the intermediate stages of tumor progression, epithelial cells undergo a vital shift in dynamics, losing intercellular adhesions. This triggers their detachment from the primary tumor, allowing subsequent invasive behavior in the nearby microenvironment. Simultaneously, mesenchymal stem cells (MCSCs) undergo a significant metamorphosis, relinquishing connections to play a crucial role in metastasis. Mesenchymal cells, possessing enhanced migratory abilities, orchestrate entry into the circulatory system. They breach the endothelial barrier using active and passive trans-endothelium migration (TEM), engaging intricately with endothelial cells. They infiltrate the mesenchymal stroma, disrupting endothelial junctions and intensifying invasiveness. Circulating tumor cells (CTCs) interact with platelet coagulation factors, producing thrombin, fostering an immune-evasive microenvironment. Neutrophils bind to CTCs, aiding immune evasion and promoting survival. CTCs decelerate, rolling along vessels, eventually halting. MCSCs adhere to the endothelium, transitioning through MET, enabling vascular traversal and colonization

Colorectal cancer (CRC) is one of the most lethal malignancies in the world. Approximately 153,020 new cases and 52,550 deaths of CRC occurred worldwide in 2022 [1]. Despite advancements in the detection and diagnosis of CRC, metastasis is still a primary cause of CRC deaths [2]. The metastatic cascade is a lengthy and laborious process. However, regardless of their final destinations, the early steps of cancer metastasis are generally similar. The epithelial-mesenchymal transition (EMT) is a well-defined process that allows cancer cells to escape the basement membrane surrounding the primary tumor and thrive at the secondary metastatic site (Fig. 1) [3, 4]. EMT is involved in almost every aspect of tumor dissemination, and new research suggests a more intricate relationship between EMT and metastasis [5, 6]. Once EMT cells invade metastatic tissue, metastatic cancer stem cells (MCSCs) at the tumor front infiltrate neighboring tissues through the blood or lymphatic vessels. MCSCs then express thrombin to bind to platelet coagulation factors and activate SMAD and Notch pathways to preserve mesenchymal characteristics [7]. In addition, circulating tumor cells (CTCs), which can disseminate as single cells or clusters, recruit platelets and neutrophils to avoid anoikis and elude immune surveillance in the bloodstream [8, 9]. When reaching secondary locations, cancer cells may become dormant to adapt to the new niche environment through EMT plasticity to retain stem cell properties [8, 10, 11].

In 1889, Doctor Stephen Paget introduced the concept of the "seed and soil" hypothesis, claiming that cancer metastasis is not a random event but selectively targets specific organs [12]. This concept is commonly referred to as "metastatic organotropism" or "organ-specific metastasis" [13, 14]. CRC typically follows a pattern of organ-specific colonization, with the liver, lungs, and peritoneum frequently serving as the initial and secondary sites of metastases, respectively [15]. Given the relatively infrequent instances of metastasis to the brain, bones, or adrenal glands in CRC cases, our focus is primarily on detailing the metastatic metastases pathways to the liver, lungs, and peritoneum [16–20].

Numerous factors have been identified to play a role in metastatic organotropism. These include the layout of the circulatory system, inherent architectural and physiological characteristics of organs, intrinsic properties of cancer itself, the organ-specific microenvironment, and the interplay between cancer cells and the native immune microenvironment [21–29]. Moreover, this process is governed by various variables such as metabolic reprogramming mechanisms, tumor-related genes, tumor-derived exosomes, and the immune system, elucidating a profound internal link between cancer cells and organ-specific metastasis [30].

Following a brief overview of the cellular and molecular mechanisms of CRC organotropic metastasis, this review aims to provide a comprehensive summary of our current understanding of how CRC acquires unique features suited for survival at specific metastatic sites during migration, such as metabolic reprogramming, tumor-derived exosomes, immune microenvironment, and key signaling molecules, which determines their ability to form distal colonization. In addition, we investigate how the numerous elements contributing to CRC metastasis could eventually lead to novel therapies for this fatal disease.

## CRC metastasis: metabolic reprogramming in organ-specific manner

**Fig. 2:**
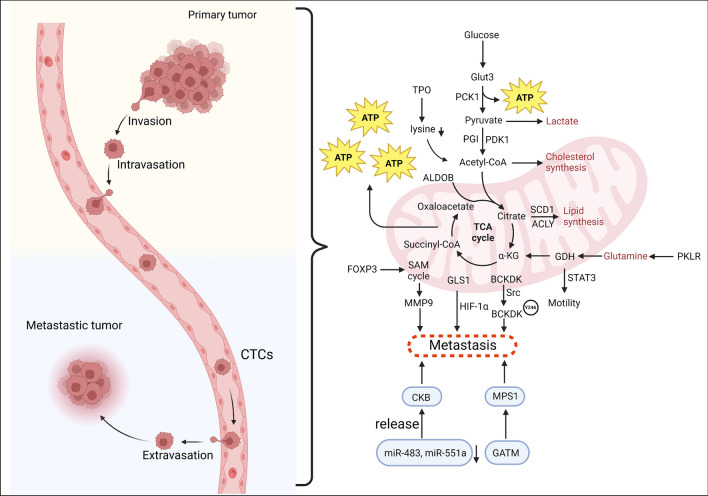
A schematic diagram shows the role of Metabolic reprogramming in the CRC metastasis. CRC cells that metastasize to liver, lung, and peritoneum need to adapt to different metabolic microenvironments to survive and grow at a distant site. During primary tumor metastasis, proliferation is the primary objective, hence there is an increase in pyruvate to lactate conversion. When a cell enters circulation, glutamine metabolism is switched on in order to produce glutathione. Increases in pyruvate and acetyl Co-A, lipid accumulation, and fatty acid uptake all contribute to the modulation of reactive oxygen species-induced damage to these circulating cells, hence enabling cell survival. Upon secondary site seeding, metastasizing cancer cells need to adapt to the metabolic microenvironments of the secondary organ, which mainly includes changes in energy and nutrient sources, organ-specific metabolites, the degree of hypoxia, and the metabolic interactions between organ-specific cells and cancer cells. The processes involve the participation of these molecules, including glucose transporter 3 (Glut3), phosphoenolpyruvate carboxykinase 1 (PCK1), pyruvate, acetyl Co-A, thyroid peroxidase (TPO), lysine, ALDOB aldolase B (ALDOB), superoxide dismutase 1 (SOD1), ATP-citrate lyase (ACLY), Citrate, α-ketoglutarate (α-KG), succinyl-CoA, oxaloacetate, glutamate dehydrogenase (GDH), pyruvate kinase isozymes R/L (PKLR), S-Adenosylmethionine (SAM), forkhead box P3 (FOXP3), matrix metalloproteinase 9 (MMP9), glutaminase 1 (GLS1), hypoxia-inducible factor 1-alpha (HIF-1α), branched-chain ketoacid dehydrogenase kinase (BCKDK), Src, creatine kinase B (CKB), monopolar spindle 1 (MPS1), guanidinoacetate N-methyltransferase (GATM)

Cancer cells must invade surrounding tissue and intravasate the vasculature to reach the bloodstream directly or via the lymphatic system. Most cancer cells die during circulation, but a few are able to colonize other organs [31]. Hence, metastatic cells require additional metabolic alterations to adapt to new environments and thrive in the microenvironment of the metastasized organ [8, 32, 33]. Indeed, metabolic abnormalities distinguish cancers from normal tissues [34]. These differences involve glucose (glycolysis, lactate production, and pentose phosphate pathway), lipids (cholesterol and acetyl-CoA), amino acids, and other nutritional metabolisms. Accumulating evidence suggests that the capacity of tumor cells to colonize distant organs and organotropism is linked to their metabolic adaptability [35–37] (Fig. 2).

Cancer metabolic reprogramming is now considered an important hallmark of cancer. Metabolic reprogramming refers to the ability of metastasizing cells to utilize metabolites to fuel different metabolic requirements of the various steps in the metastatic cascade [38, 39]. In addition, through metabolic reprogramming, cancer cells can fulfill their high-energy needs and cope with the intrinsic stress associated with a high proliferation potential [37].

Liver metabolism regulates the energy balance of the body. Maintaining blood glucose levels requires glucose consumption and production, fatty acids and ketone bodies synthesis, and protein synthesis and breakdown. To inhibit cancer cell metastasis, the liver is partitioned into metabolic regions corresponding to varied oxygen gradients to execute its metabolic functions effectively. Typically, the liver microenvironment favors cells with a high glycolytic profile and low-oxygen adaptations. Therefore, for patients with colorectal liver metastases (CRLM), metastasizing cancer cells from other organs must overcome this hypoxic barrier and adapt to the liver environment [40–43].

This phenomenon is exemplified by a plethora of studies that have demonstrated the preferential utilization of amino acid metabolism by colorectal cancer cells for their proliferation within hepatic tissues. For example, glutamine dehydrogenase (GDH) is required for glutamine metabolism since it converts glutamate to α-ketoglutarate (α-KG) in the presence of inadequate glucose or hypoxia. Meanwhile, GDH increased CRC cell motility through STAT3-mediated EMT induction, implicating GDH in CRC's metastatic and aggressive biology [44]. Additionally, colorectal cancer liver metastasis facilitating by upregulating Glutaminase 1 (GLS1) in a hypoxia-inducible factor 1α (HIF-1α)-dependent manner [45]. Wu et al*.* revealed that lysine catabolism was indispensable for liver colonization by CD110 + tumor-initiating cells (TICs). The thrombopoietin (TPO) promoted metastasis of CD110 + TICs to the liver by activating lysine degradation. In contrast, lysine catabolism generated acetyl-CoA, which could activate Wnt signaling to promote self-renewal of CD110 + TICs by triggering tyrosine phosphorylation of LRP6. Lysine catabolism also generates glutamate, which modulates the redox status of CD110 + TICs to promote liver colonization and drug resistance [46]. The essential amino acid methionine is an emergent feature of cancer metabolism. The methionine cycle transforms methionine into S-adenosyl-methionine (SAM), the fundamental methyl donor. Wang et al*.* showed that the overexpression of FOXP3 facilitated MMP9 expression through the SAM cycle to modulate CRC liver metastasis [47].

Glycolytic metabolism is also an indispensable factor in the process of liver metastasis in colorectal cancer. Qin et al*.* found that the knockdown of PDK1, a glycolysis enzyme, dramatically reduced CRC liver metastases. PDK1 knockdown enhanced reactive oxygen species (ROS) levels in anoikis, which elevated anoikis and reduced liver metastases [48]. Bu et al*.* demonstrated that colon cancer metastasizing in the liver of mice was fueled by the upregulation of the enzyme fructose-bisphosphate aldolase B (ALDOB), which is involved in fructose metabolism. During the growth of tumor cells, metastatic liver cells upregulated the enzyme aldolase B (ALDOB), which improved fructose metabolism and supplied fuel for critical pathways of central carbon metabolism [49]. Phosphoglucose isomerase (PGI) is a ubiquitous cytosolic enzyme critical in glycolysis. Tsutsumi et al*.* showed that the overexpression of PGI strongly contributed to the aggressive phenotype of human colon cancer [50]. Liver metastasis hyperacetylated isocitrate dehydrogenase 1 (IDH1) K224 by inhibiting sirtuin-2 (SIRT2) in hypoxia-inducible factor 1α-dependent SRC transcription. This mechanism has been demonstrated to be essential for the growth of liver metastases as genetic deacetylation boosts its enzymatic activity, and the generation of α-KG entirely inhibits its invasion and migration [51]. Phosphoenolpyruvate carboxykinase (PEPCK or PCK) catalyzes the first rate-limiting step in the hepatic gluconeogenesis pathway, which is responsible for glucose homeostasis. Yamaguchi et al*.* revealed that PCK1 promoted CRC liver metastatic colonization. Mechanism studies showed that PCK1 promoted pyrimidine nucleotide synthesis, which supported cancer cell proliferation during hypoxia [52]. In a similar vein, PKLR operates as a homotetramer and triggers the generation of phosphoenolpyruvate from pyruvate and ATP. This action fosters the spread of colon cancer cells to the liver without contributing to basal growth in culture. Investigations into the underlying mechanisms revealed that PKLR sustains cell viability within the tumor's core in the presence of high cell density and oxygen deprivation. Furthermore, it is essential for preserving glutathione levels, a key endogenous antioxidant, in order to bolster cancer cell survival [53].

The liver is also involved in creatine metabolism. Loo et al*.* showed that by downregulating miR-483 and miR-551a, liver metastases arising from CRC stimulated the release of brain-type creatine kinase (CKB) into the microenvironment. The reinjection of metastatic cells into mice reduced their ability to form liver colonies when the expression of CKB or the creatine transporter SLC6A8 was diminished [54]. Moreover, creatine supplementation in vitro or synthesis in vivo enhanced cancer metastasis. In this study, cell experiments, multiple mouse model experiments, and analysis of patient samples entirely proved that increased intratumoral creatine levels caused by exogenous supplementation or autogenous synthesis mediated by GATM could upregulate the expression of Slug and Snail by MPS1 activation of Smad2/3, thereby promoting tumor liver metastasis [55].

The lungs contribute to appropriate respiratory system function and cellular homeostasis through numerous metabolic pathways. Importantly, drug and toxin metabolism in the lungs regulates the acid–base balance within the body. The lungs are full with mitochondria, the energy-generating organelles within cells that play a pivotal role in facilitating ATP production via cellular respiration [23]. Additionally, lung enzymes support energy production by metabolizing carbohydrates, fats, and proteins. However, due to their exposure to both oxygen and toxins, the lungs are highly susceptible to oxidative stress, requiring a robust defense mechanism against ROS. In order to colonize the lungs successfully, cancer cells, such as colorectal pulmonary metastases (CRPMs), must possess the ability to combat oxidative stress in the lung microenvironment [56].

Lipid metabolism plays a crucial role in the pathogenesis of lung metastasis from colorectal cancer and is closely associated with enhanced resistance to oxidative stress in conjunction with colorectal cancer. A mechanism of lipid metabolism implicated in lung metastasis was the overexpression of ATP-citrate lyase (ACLY), a protein in the initial rate-controlling step of lipid synthesis, which was elevated in CRC and played an important role in CRC lung metastasis. Specifically, lung metastases from colorectal tumors upregulated the expression of ACLY, hence increasing cell lipid production and lung metastasis [57]. Another lipid metabolism mechanism that plays a significant role in lung metastasis is upregulated stearoyl-CoA desaturase 1 (SCD1) expression. In vitro experiments showed that SCD1 promoted EMT. The author further investigated the revelation that SCD1 increased MUFA levels, regulating fatty acid composition. Furthermore, in response to high glucose, carbohydrate response-element binding protein (ChREBP) enhanced the progression of CRC via SCD1. Mechanistically, hyperglycemia-SCD1-MUFA caused CRC cell migration and invasion via PTEN regulation [58].

Moreover, changes in cellular glycolytic metabolism can enhance antioxidant capacity, which may contribute to the metastasis of colorectal cancer. Indeed, the activation of the Glut3-YAP signaling pathway in CRC metastatic cells functions as a master stimulator to alter cancer metabolism, allowing for lung-preferred metastasis. In addition, inhibiting Glut3 in CRC cells significantly decreased their metastatic lung potential. The authors speculated that this metastatic advantage was due to the activation of YAP, which transactivates Glut3 and regulates a collection of glycolytic genes, particularly the phosphorylation of PKM2 was elevated in metastatic CRC and interacted with YAP to boost Glut3 expression. Overall, the YAP-Glut3 signaling pathway boosted lung metastasis via increased cancer cell metabolic reprogramming [59].

Next, we discuss branched-chain α-keto acid dehydrogenase kinase (BCKDK), a crucial enzyme in branched-chain amino acid (BCAA) metabolism. At first glance, this result may appear consistent, considering that BCAAs can be used to synthesize proteins or metabolize tumors for energy. However, at the cellular level, direct supplementation with BCAAs failed to promote CRC cell migration and invasion, demonstrating that BCKDK acted prometastatically in CRC cells in a BCAA-independent manner. Combined with phosphoproteomics analysis, they identified a novel upstream regulator of BCKDK, Src, that phosphorylated BCKDK at the tyrosine 246 (Y246) site, enhancing CRC cell migration, invasion, and EMT [60]. Also, Homeobox A13 (HOXA13) plays a role in expediting CRC lung metastasis by generating a significantly conserved family of transcription factors, which oversee an array of cellular functions such as cell growth, differentiation, cell death, receptor signal transmission, the formation of new blood vessels, and metabolic activity throughout the embryogenesis process [2]. Interestingly, despite containing an abundance of oxygen in the lung, some CRC metastases resist strong oxidative stress, probably because they have the metabolic adaptability to survive in high-oxygen environments [61].

Taken together, despite its underappreciation in clinical trials and practice, metabolic reprogramming is necessary for cancer cells to survive, adapt, and thrive in a remote metastatic area.

## The role of exosomes in organ-specific CRC metastasis

**Fig. 3:**
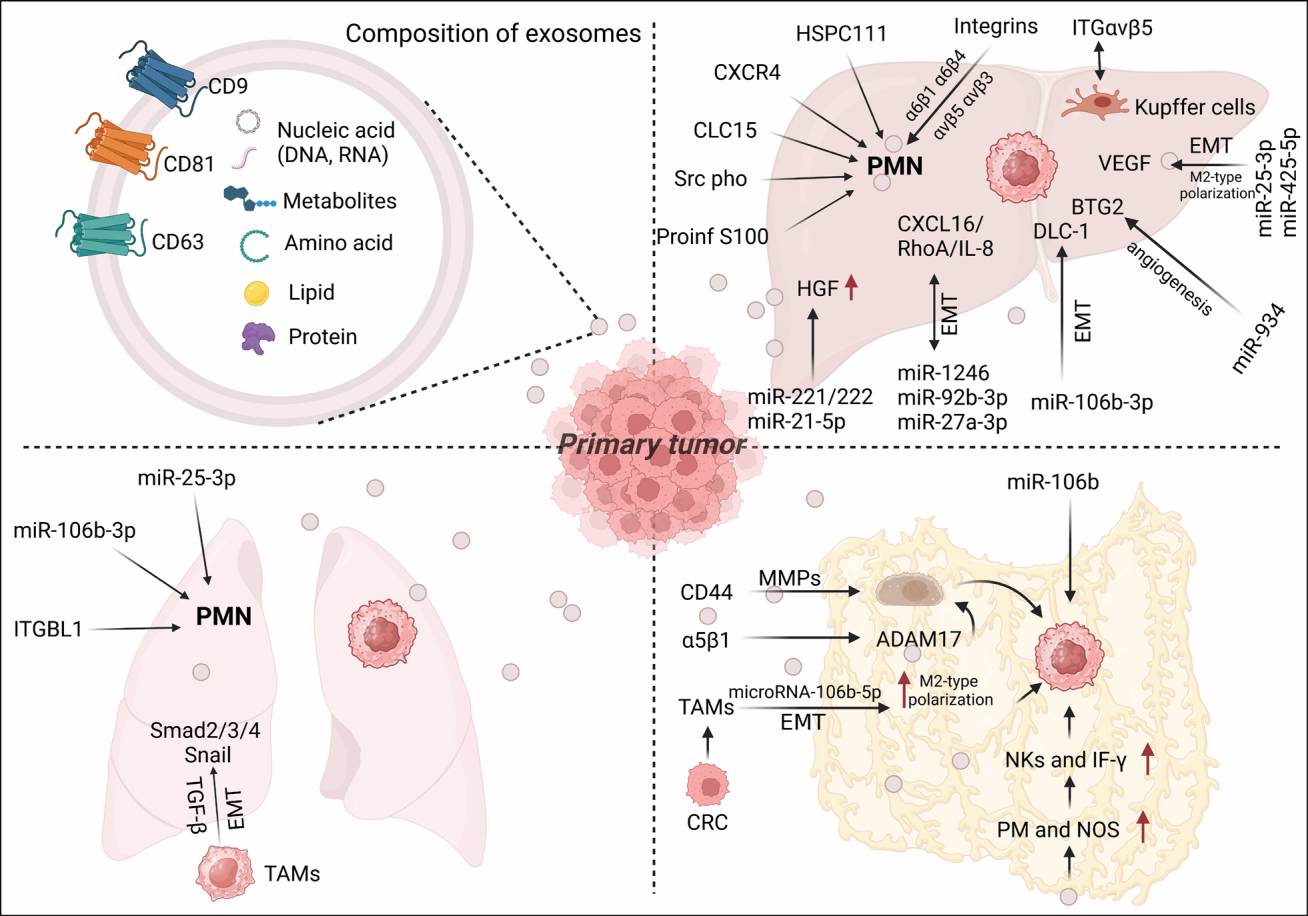
A schematic illustration of the CRC-derived exosome mechanisms for CRC metastasis. CRC-derived exosomes represent extracellular vesicles produced by CRC cells that transport various substances such as nucleic acids, proteins, lipids, and metabolites throughout the tumor microenvironment. These exosomes play a pivotal role in the formation of PMNs. Primary tumor cells release exosomes that can disseminate to distant metastatic sites and modulate local cells within the pre-metastatic microenvironment through diverse signaling pathways, such as upregulation of pro-inflammatory gene expression and immunosuppressive cytokine secretion. Moreover, these exosomes also participate in regulating the immune response, promoting increased angiogenesis, and facilitating cellular epithelial-mesenchymal transition (EMT). Their involvement is particularly prominent in liver, lung, and peritoneal metastases. In these metastatic sites, exosomes act as crucial mediators between cancer cells and the surrounding microenvironment, thereby facilitating the reorganization of the secondary site to enable successful tumor colonization

Extracellular vesicles are nanosized vesicles (30–150 nm in diameter) that play vital roles in intercellular communications. They carry diverse biomolecules, such as proteins, glycans, lipids, metabolites, RNA, and DNA, in human biofluids, including blood, urine, and cerebrospinal fluid [62, 63]. Transmembrane 4 superfamily tetraspanins and integrins (ITGs) are the main molecules on the surface of exosomes [64]. CD9, CD63, and CD81 are commonly utilized as specific markers for exosomes [65, 66]. Tumor-derived exosomes (TDEs) are essential in affecting immune responses, cell migration, proliferation, differentiation, and tumor invasion [67] (Fig. 3, Table 1).

**Table 1:** Targets for organotropic metastasis in this review

Organotropic site	Target	Role	Mechanism	Authors
Liver metastasis	*GDH*	Promotes	STAT3-mediated EMT induction	Liu et al. [43]
	*GLS1*	Promotes	HIF-1 promotes GLS1 expression	Xiang et al. [44]
	*TPO*	Promotes	TPO enhances hepatic metastasis of CD110 + TICs by activating lysine degradation	Wu et al. [45]
	*FOXP3*	Promotes	Potential to play a role in cancer progression and metastasis	Wang et al. [46]
	*PDK1*	Promotes	PDK1 knockdown increased reactive oxygen species	Qin et al. [47]
	*ALDOB*	Promotes	Metastatic cells in the liver upregulate the ALDOB, which enhances fructose metabolism	Bu et al. [48]
	*PGI*	Promotes	Overexpression of PGI contributes to the aggressive phenotype of human colon cancer	Tsutsumi et al. [49]
	*SIRT2*	Promotes	Liver metastasis involves IDH1 K224 hyperacetylation by inhibiting SIRT2 through HIF-1α-dependent SRC transcription, promoting invasion and migration	Wang et al. [50]
	*PEPCK*	Promotes	PCK1 enhances pyrimidine nucleotide production, which facilitates cancer cell Development in the context of hypoxia	Yamaguchi et al. [51]
	*PKLR*	Promotes	PKLR promotes cell survival in the tumor core in high cell density and hypoxia	Nguyen et al. [52]
	*CKB*	Promotes	By downregulating miR-483 and miR-551a, CKB release into the microenvironment is stimulated, promoting liver colonies	Loo et al. [53]
	*GATM*	Promotes	Elevated intratumoral creatine levels or GATM-mediated synthesis can enhance Slug and Snail expression via MPS1-activated Smad2/3, promoting liver metastasis	Zhang et al. [54]
	*CXCR4*	Promotes	Exosomes may promote colorectal cancer metastasis by recruiting CXCR4-expressing stromal cells to form a permissive metastatic microenvironment	Wang et al. [71]
	*Exosomal HSPC111*	Promotes	Mediated pre-metastatic niche formation	Zhang et al. [72]
	*ITGαvβ5*	Promotes	ITGαvβ5 preferentially adhered to Kupffer cells, enhancing hepatic tropism	Hoshino et al. [70]
	*Exosomal* *miR-221/222*	Promotes	Activates hepatic hepatocyte growth factor (HGF) in CRC exosome	Tian et al. [75]
	*Exosomal* *miR-21-5p*	Promotes	Caused a pro-inflammatory phenotype and liver metastasis of cancer via the miR-21-Toll-like receptor 7-IL-6 axis	Shao et al. [76]
	*Fn-derived miR-1246,92b-3p,27a-3p*	Promotes	Facilitate the liver metastasis of uninfected cells	Guo et al. [77]
	*miR-139-3p* *miR-193a* *let-7 g*	Promotes	Plasma exosomal was used to monitor CRC metastasis in real-time	Liu-cho et al. [78, 79]
	*miR-253p,130b-3p,425-5p,* *193a*	Promotes	Potential to play a role in cancer progression and metastasis	Liu, Wanget al. [80, 81]
	*Exosomal miR-934*	Promotes	Enhanced M2 macrophage polarization in CRC cells by downregulating PTEN levels and activating the PI3K/AKT pathway	Li et al. [82]
	*Exosomal miR-1246*	Promotes	Mutant p53 CRC can transduce macrophages into M2-like macrophages with the help of exosomal miR-1246	Cooks et al. [113]
	*HGF/cMet, PRL3, L1CAM, CXCR4, CAFs, Trop-2, ZFP57*	Promotes	Those key candidate genes associated with colon cancer liver metastasis	Trusolino ~ Shoji et al. [148–160]
Lung metastasis	*ACLY*	Promotes	Increased expression of ACLY may enhance cell lipid production and lung metastasis	Wen et al. [56]
	*SCD1*	Promotes	Hyperglycemia-SCD1-MUFA caused CRC cell migration and invasion via PTEN regulation	Ran et al. [57]
	*Glut3*	Promotes	Stimulation of the Glut3-YAP signaling pathway acts as a master activator to change the cancer metabolism	Kuo et al. [58]
	*BCKDK*	Promotes	Phosphorylated BCKDK at the tyrosine 246 (Y246) site, enhancing CRC cell invasion	Tian et al. [59]
	*HOXA13*	Promotes	Expedited CRC lung metastasis via performing	Qiao et al. [60]
	*Exosomes* *(MiR-25-3p MiR-106b-3p ITGBL1)*	Promotes	CRC cells induce premetastatic niche formation by secreting exosomes to promote CRC lung metastasis	Song, Wang, Ji et al. [42, 83, 84]
	*TAM secreting* *(TGF-β)*	Promotes	Induces EMT by activating the Smad2/3/4 Snail pathway	Shen et al. [85]
	*KRAS mutation*	Promotes	Predisposition to lung metastasis	Kim,Tie,Cho et al. [161–163]
	*REG1B, TGM6, NTF4, PNMA5, and HOXC13*	Promotes	Those key candidate genes associated with colon cancer lung metastasis	Zhou et al. [164]
Peritoneal metastasis	*CD44*	Promotes	CD44-enriched vesicles induce the secretion of matrix metalloproteinases (MMPs), compromising mesothelial barrier integrity and facilitating cancer cell invasion	Nakamura et al. [87]
	*α5β1* *ADAM17*	Promotes	Interactions between integrin α5β1 on CRC cells and its ligand ADAM17 on exosomes facilitate CRC-derived exosome binding and uptake, promoting cancer cell invasion	Cardenes et al. [88]
	*MicroRNA-106b-5p*	Promotes	MicroRNA-106b-5p promotes the polarization of M2 macrophages by inhibiting PDCD4, thereby mediating the metastasis of colorectal cancer (CRC) cells	Yang et al. [88, 89]
	*Tumor-derived exosomes*	Promotes	Exosomes may facilitate colorectal cancer peritoneal metastasis progression by modulating immune responses, including increased macrophage numbers and enhanced natural killer cell activation	Tokuda et al. [90]
	*Tumor-derived exosomes*	Promotes	Exosomes may undergo alterations during the peritoneal metastasis process, thereby influencing the tumor microenvironment and interactions	Vallejos et al. [91]
	*IL-4, IL-10, TGF-β, M-CSF, andprostaglandin E2*	Promotes	By promoting M2 cell polarization, tumor progression is mediated	Yin, Novak, Schreiber et al. [129–131]
	*TGF-β, TNF-β, and IGF1*	Promotes	By promoting *CAFs*, tumor progression is mediated	Koliaraki et al. [140]
	*FBXW7*	Inhibits	Inhibition of distant metastasis in colorectal cancer	Mlecnik, Stein, Stein et al. [165–167]
	*MUC1*	Promotes	By promoting epithelial-mesenchymal transition (EMT) while suppressing cell apoptosis	Schroeder et al. [169]
	*TIMP-2, IGF-1, and HIF-1A*	Promotes	Increased expression in clones of peritoneal metastasis from colorectal cancer	Lemoine, Varghese et al. [25, 170]
	*CMS4 subtype*	Promotes	Significant enrichment of CMS4 in primary tumors with peritoneal metastasis	Ubink, Laoukili et al. [171, 172]

Tumor cells influence target organs prior to invasion through premetastatic niches (PMNs), which contribute to organ-specific metastasis formation [68, 69]. Complex signals are exchanged between target organs and TDEs during the development of PMNs [70]. Hoshino et al*.* demonstrated in 2015 that exosomes precisely influenced the organogenesis of cancer cells, driving them to their desired alignment [71]. Another study by Wang et al*.* demonstrated that exosomes from colorectal cancer cells expressing CXCR4 affect liver metastasis of colorectal cancer cells, suggesting that exosomes may promote colorectal cancer metastasis by recruiting CXCR4-expressing stromal cells to form a permissive metastatic microenvironment [72]. Likewise, exosome HSPC111 derived from colorectal cancer upregulates acetyl coenzyme A levels by phosphorylating ATP-citrate cleavage enzyme (ACLY), leading to an accumulation of acetyl coenzyme A that subsequently enhances H3K27 acetylation. This process ultimately increases CXCL5 expression and secretion, thereby mediating hepatic colonization of colorectal cancer through modulation of the hepatic microenvironment [73]. In addition, tumor cell attachment and extracellular matrix molecules like integrins affect distant metastasis [74]. Integrins, adhesion molecules, mediate cell–cell and cell–extracellular matrix interactions. It has been demonstrated that tumor-derived exosomes and integrin patterns, especially α6β1, α6β4, αvβ5, and αvβ3, which are linked to ECM molecules and certain cell types in target organs, might merge with organ-specific cells and activate Src phosphorylation and proinflammatory S100 expression to establish a PMN [71]. Additionally, novel exosome adhesion molecules have been found to contribute to organotropism, and different organs have unique characteristics. For instance, exosomes expressing ITGαvβ5 preferentially adhered to Kupffer cells, enhancing hepatic tropism [71]. Kupffer cells also contribute to liver metastasis by increasing exosome uptake via the activation of hepatic stellate cells [75].

Additionally, certain exosomes containing proteins and microRNAs have diagnostic and prognostic potential for predicting metastatic sites in patients with colorectal cancer. For instance, researchers observed that miR-221/222, which was significantly elevated in serum exosome samples from CRC patients with liver metastases, activated hepatic hepatocyte growth factor (HGF) by suppressing SPINT1 expression in CRC exosomes, making CRC more aggressive [76]. Similarly, a recent study by Shao et al*.* found that exosomes carrying miR-21-5p from CRC cells promoted a pro-inflammatory phenotype and hepatic metastasis by activating the miR-21-Toll-like receptor (TLR) 7-IL-6 signaling axis [77]. Interestingly, miR-27a-3p, miR-1246, and miR-92b-3p derived from *Fusobacterium nucleatum (Fn)*-infected CRC cells have been found to facilitate liver metastasis. Specifically, exosomes transferred miR-1246/92b-3p/27a-3p and CXCL16/RhoA/IL-8 from Fn-infected to non-infected cells, thereby enhancing the in vitro migratory capacity of recipient cells and promoting tumor metastasis in vivo [78]. Recently, plasma miR-139-3p, miR-193a, and let-7 g were used to monitor CRC metastasis in real-time [79, 80]. Exosomes containing miR-130b-3p, miR-425-5p, miR-25-3p, and miR-934 may also play roles in cancer progression and metastasis. However, their mechanisms are distinct, as exosomal miR-106b-3p promotes epithelial-mesenchymal transition (EMT) and targets the tumor suppressor gene DLC-1 to regulate hepatocellular carcinoma metastasis. Serum exosomes containing miR-25-3p and miR-425-5p promote epithelial-mesenchymal transition (EMT) and secrete vascular endothelial growth factor (VEGF), thereby facilitating tumor metastasis through activation of the PI3K/Akt signaling pathway that regulates PTEN-induced macrophage M2-type polarization. Furthermore, exosome-mediated delivery of miR-934 enhances angiogenesis in vascular endothelial cells by directly binding to b-cell translocation gene 2 (BTG2) [81–83].

In addition to liver metastasis, exosomes also play an essential role in CRC lung metastasis. The tumor microenvironment (TME) and tumor-derived exosomes can induce the formation of PMN and increase the risk of CRC lung metastasis. Multiple studies discuss the formation of a microenvironment by exosomes prior to lung metastasis in colorectal cancer. For instance, CRC cells secrete exosomes that contain miR-25-3p, miR-106b-3p, and ITGBL1 to induce the formation of PMNs and promote lung metastasis in CRC [42, 84, 85]. Similarly to extracellular vesicles, tumor-associated macrophages (TAMs), the main infiltrating inflammatory cells in the TME, secrete cytokines that mediate distant metastasis and participate in CTC formation. Cai et al*.* demonstrated that TAMs induce EMT by activating the Smad2/3/4 Snail pathway through TGF-β secretion, thereby promoting distant lung metastasis in CRC [86].

Analogous to solid tumors, malignant cells in peritoneal metastasis (PM) are situated within a tissue matrix comprising an array of cellular and acellular constituents, which facilitate intercommunication. Cellular components encompass immune cells, vascular cells, and stromal cells, whereas acellular components comprise extracellular matrix (ECM) structures, extracellular vesicles (EVs), as well as collagen, laminin, fibronectin, and proteoglycans. Notably, as an acellular component, EVs play a pivotal role in mediating communication between unbound tumor cells and target organs, thereby promoting the metastatic process [87]. Recent studies have emphasized the role of exosomes in PM pathogenesis. Extracellular vesicles derived from colorectal cancer cells are rich in the cell surface glycoprotein CD44. When introduced to mesothelial cells (MC), these CD44-enriched vesicles induce the secretion of matrix metalloproteinases (MMPs), compromising mesothelial barrier integrity and facilitating cancer cell invasion [88]. Concurrently, research has discovered that interactions between integrin α5β1 on colorectal cancer (CRC) cells and its ligand *microRNA-106b-5p* on exosomes regulate the binding and uptake of CRC-derived exosomes. This process promotes cancer cell adhesion to peritoneal mesothelial cells (PMC) in peritoneal organs and enhances cancer cell invasion through the mesothelial barrier and underlying matrix [89]. Exosomes also contribute to metastasis by suppressing the immune system. For example, CRC cells induce M2 polarization of tumor-associated macrophages (TAMs) through direct transfer of exosomes, which increases the levels of microRNA-106b-5p. The activation of this microRNA inhibits PDCD4. Activated M2 macrophages subsequently promote EMT-mediated CRC cell migration, invasion, and metastasis. Clinically, elevated exosomal miR-106b expression is associated with CRC progression [90]. Moreover, studies have shown that tumor-derived exosomes can induce an increase in peritoneal macrophage populations and the expression of inducible nitric oxide synthase, as well as enhance activated natural killer cell and interferon-gamma expression. These findings imply that exosomes may facilitate colorectal cancer peritoneal metastasis progression by dampening immune responses [91]. In another pilot study, researchers employed mass spectrometry-based proteomics to reveal a distinct enrichment of specific tumor-derived exosomes in peritoneal metastasis compared to in situ tumors. This suggests that the proteomic composition of exosomes may undergo alterations during the peritoneal metastasis process, thereby influencing the tumor microenvironment and interactions [92].

In summary, exosomes are essential in forming PMNs and dispersal CRC cells to distant sites.

## The interplay between the immune system and organ-specific metastasis in CRC

Human immune cells (innate/adaptive immune cells) can recognize and eliminate tumor cells as an important cancer defense. The innate immune system consists of macrophages, neutrophils, monocytes, eosinophils, basophils, myeloid cells, and natural killer cells, while the adaptive immune system contains B cells, CD8^+^ T cells, and CD4^+^ T cells. These immune cells provide innate immunity against pathogens to maintain host homeostasis [93]. However, metastatic immune evasion at the primary tumor site requires innate/adaptive immune subversion. Therefore, the capacity of cancer cells to co-opt immune processes to help in these steps and to escape immune detection is essential for effective metastasis [94, 95].

**Fig. 4:**
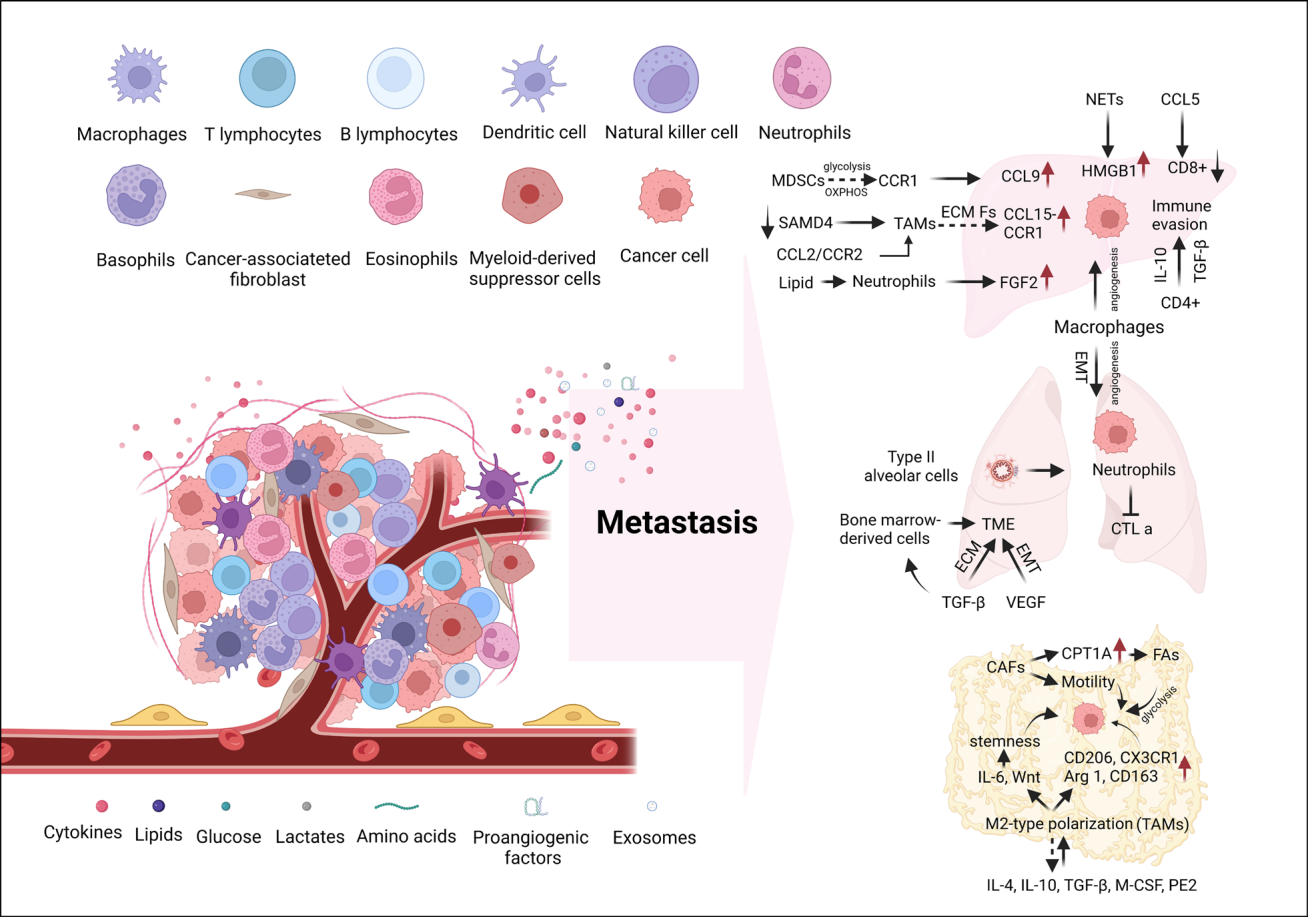
A diagrammatic representation of the interplay among immune cells and CRC cells unveils the molecular mechanism underpinning CRC metastasis. The inborn immune system is comprised of macrophages, neutrophils, monocytes, eosinophils, basophils, and natural killer cells, while the acquired immune system includes B cells, CD8 + T cells, and CD4 + T cells. When the innate immune system becomes imbalanced, it can contribute to the metastasis of CRC. One critical aspect contributing to the promotion of CRC metastasis is the secretion of molecules such as Chemokine Receptor (CCR), Chemokine Ligand (CCL) and Interleukin by immune cells. These molecules play a pivotal role in creating a microenvironment conducive to EMT of tumor cells. Moreover, these molecules may suppress the immune surveillance mechanisms that would otherwise identify and eliminate cancer cells. In addition to chemokines, various components within the tumor microenvironment contribute to immune evasion and thus facilitate metastasis. Exosomes, for instance, tiny vesicles released by tumor cells, can carry immunosuppressive molecules that dampen the activity of immune cells. Cancer-associated fibroblasts (CAFs) and tumor-associated neutrophils (TANs) also play a role in creating an immunosuppressive microenvironment

Immune cells interact with cancer cells, influencing their metastatic potential, including initiation, survival, growth, and metastasis [96, 97] (Fig. 4). The liver serves as a crucial immune organ within the body. A crucial factor in CRLM is the immune microenvironment. The roles of tumor-associated macrophages (TAMs), regulatory T cells (Tregs), and tumor-associated neutrophils (TANs) are critical during tumor metastasis progression [98]. In murine colon cancer models, the chemokine receptor CCR1 on myeloid cells instigates them to accumulate in the liver's microenvironment, leading to the secretion of its ligand, CCL9 (akin to CCL15 in humans) in cancer cells, and then potentiates metastasis, indicating that CCR1-positive myeloid cells may participate in the initial phases of hepatic metastases [99]. In addition, TANs recruitment can be triggered when SMAD4 is lost, promoting CCL15-CCR1 expression [100]. Another study conducted by Rodero et al. demonstrated that the expression of CCR1, both hematopoietic and non-hematopoietic, promotes angiogenesis in metastatic cancer cells [101]. Neutrophils infiltrated both mouse and human liver metastases from CRC and significantly expressed FGF2, indicating polarization of neutrophils by the tumor microenvironment [102]. And neutrophil extracellular traps (NETs) promote cancer cell migration and invasion during stress responses by releasing HMGB1 [103]. Moreover, as a crucial factor in the metabolic response of colorectal cancer, lipid accumulation can facilitate neutrophil infiltration and subsequently enhance the likelihood of liver metastasis in patients with colorectal cancer [104]. T lymphocytes govern organotropic metastasis. Flow cytometry analysis of peripheral blood and tumor/paraneoplastic tissues from colorectal cancer (CRC) patients and healthy controls revealed the accumulation of LAP + CD4 + T cells in the tumor microenvironment, with immune evasion mediated by IL-10 and TGF-β promoting tumor metastasis [105]. CCL5 depletion promoted CD8^+^ T-cell accumulation in CRC mice models, inhibiting tumor development and metastasis [106]. Additionally, T cells are important immune cells in the tumor microenvironment because they have a dual effect on oncogenesis and cancer progression [107]. For instance, cholesterol metabolism in T cells impacts cancer progression. On the one hand, they inhibit acetyl-CoA acetyltransferase 1 (ACAT1), stimulate the formation of immune synapses, and boost the antitumor ability of CD8^+^ T cells [108]. On the other hand, cholesterol accumulation in TAMs can increase fatty acid intake, lipid peroxidation, and ferroptosis in CD8^+^ T cells, leading to CD8^+^ T-cell dysfunction and antitumor immunity. Furthermore, macrophages participate in angiogenesis by producing proangiogenic factors and cytokines [109]. The interplay between metabolic reprogramming, exosomes, macrophages and cancer cells can facilitate the progression of cancer metastasis [110–112]. For example, exosome-mediated transfer of miRNA-934 has been shown to enhance macrophage polarization towards an M2 phenotype in CRC cells by reducing PTEN expression levels and activating the PI3K/AKT signaling pathway [113]. The presence of exosomal miR-1246 in mutant p53 CRC assists in the transformation of macrophages into M2-like cells [114]. Immunosuppressive myeloid-derived suppressor cells (MDSCs) originate from bone marrow progenitor cells and immature myeloid cells, and tumor-related MDSCs upregulate anaerobic glycolysis and OXPHOS expression also facilitate cancer metastasis [115]. Furthermore, TAMs release ECM remodeling factors and proteolytic enzymes that degrade ECM proteins, such as MMPs, allowing tumor cells to migrate [116]. CCL2/CCR2 chemokine treatment reduces TAM infiltration at metastatic sites and makes tumor T cells more sensitive to cancer [117]. Moreover, as IL-6 activates the IL-6 receptor (IL-6R), miR-34A expression is inhibited, increasing the invasion and metastasis of CRC via EMT [118], and IL-33 induces liver metastasis via TME [119].

The lung parenchymal microenvironment encompasses an assortment of immune cell types that serve essential protective functions against detrimental airborne pathogens, toxic compounds, and inflammatory agents [120, 121]. It is crucial to note that persistent inflammatory states, such as chronic obstructive pulmonary disease (COPD) and tobacco smoking, can alter this microenvironment, increasing vulnerability to primary tumor formation and the genesis of pre-metastatic niches [122]. Lung metastases, specifically CRPMs, exhibit a distinctive inclination towards a more immune-responsive TME than metastases located in other organs, including the brain, liver, and bone. Intriguingly, lung metastases from diverse primary malignancies (encompassing CRC) possess a preponderance of genes associated with antigen presentation and immune effector cells, such as cytotoxic T lymphocytes (CTLs) and B-lineage cells. These metastases also exhibit a decreased presence of suppressor cells and the increased expression of immune checkpoint proteins, notably PD-L1 and CTLA-4, which may contribute to their ability to circumvent immune recognition [123].

The intricate process of lung metastasis development from extra-thoracic neoplasms entails primary tumors releasing extracellular vesicles and pro-metastatic factors, including Transforming Growth Factor-beta (TGF-β) and Vascular Endothelial Growth Factor (VEGF). These elements not only contribute to the reorganization of the Extracellular Matrix (ECM), the facilitation of Epithelial-Mesenchymal Transition (EMT), and the enablement of invasion into the systemic circulation, but also participate in the recruitment of bone marrow-derived cells to the microenvironment [124]. Following this, circulating tumor cells permeate pulmonary tissue, while type II alveolar cells enlist neutrophils to restrain CTL activity and cooperate with fibroblasts to incorporate tumor cells within the lung parenchyma. Additionally, macrophages present in the metastatic niche bolster tumor cell survival and proliferation. In conclusion, the established metastatic niche fosters tumor cell expansion, instigates Mesenchymal-Epithelial Transition (MET), and augments angiogenesis [125].

As previously discussed, the presence of ascites is associated with peritoneal metastatic cancer. The ascitic fluid contains a sophisticated blend of immune cells, tumor cells, cytokines, and various cellular constituents, giving rise to a remarkably intricate microenvironment for peritoneal metastasis. Within this fluid milieu, immune and cancer cells can engage in direct contact as the ascites circulate, facilitating the interaction of cytokines and proteins that contribute to the complex dynamics of this liquid microenvironment. Within this microenvironment, macrophages represent the most abundant cell population, accounting for 45–50% of the total cells [126]. These macrophages release an array of inhibitory cytokines that orchestrate a favorable immune milieu for cancer cell survival and peritoneal invasion. This is achieved by promoting tumor angiogenesis, impairing T cell function, and inducing the differentiation of regulatory T cells (Tregs). This complex interplay between macrophages and the immune landscape is intimately associated with peritoneal metastasis in cancer, underscoring the pivotal role macrophages play in this context [127, 128]. In the early stages of tumor development, macrophages exhibit a pro-inflammatory phenotype, characterized by the secretion of pro-inflammatory cytokines and reactive oxygen species, which aids in the elimination of pathogens. Moreover, these cells participate in antigen presentation, eliciting T-cell responses that target tumor cells [129]. However, as tumors progress, the tumor microenvironment becomes enriched with cytokines, such as IL-4, IL-10, TGF-β, M-CSF, and prostaglandin E2, promoting the polarization of macrophages towards the M2 phenotype. This phenotypic switch is accompanied by the upregulation of markers, such as CD206, CX3CR1, Arginase 1, and CD163. These M2 macrophages, commonly referred to as tumor-associated macrophages (TAMs), predominantly exert immunosuppressive functions and play critical roles in tumor growth, invasion, metastasis, and drug resistance [130–132].

In general, TAMs have been shown to stimulate tumor growth and metastasis by facilitating angiogenesis, initiating pre-metastatic niches, and dampening immune responses [133]. Notably, studies have demonstrated that TAMs can induce the formation of specialized multicellular spheroids by enveloping peritoneal cancer cells. Positioned at the center of these spheroids, TAMs secrete EGF to promote tumor cell proliferation and peritoneal invasion [130]. Furthermore, evidence suggests that cancer stem cells (CSCs) can form multicellular spheroids around TAMs, maintaining their stemness through the activation of IL-6 and WNT signaling pathways and ultimately promoting tumor drug resistance and peritoneal invasion [134]. Recent findings also implicate TAMs in inducing invasive behavior and epithelial-mesenchymal plasticity in colon cancer cells [135, 136]. Beyond their impact on tumor cells, TAMs can directly or indirectly modulate T cell function through intricate mechanisms involving multiple signaling pathways. By secreting TNF-α and IL-10, TAMs upregulate their own PD-L1 expression, inducing T cell dysfunction and directly suppressing T cell proliferation and cytotoxicity. Consequently, this promotes tumor growth and is associated with a poorer prognosis [133, 137, 138].

In the peritoneal microenvironment, another crucial cell type is cancer-associated fibroblasts (CAFs), which facilitate tumor (peritoneal) metastasis and immune evasion [139, 140]. The primary source of CAFs in peritoneal metastasis is submesothelial fibroblasts, whose phenotypic diversity is driven by various mediators such as transforming growth factor (TGF)-β, tumor necrosis factor (TNF)-β, and insulin-like growth factor I (IGF1) [141]. In vitro and xenograft mouse models have demonstrated that submesothelial fibroblasts create a permissive environment for colorectal peritoneal metastasis invasion and dissemination [142]. Recent research on colorectal cancer peritoneal metastasis indicates that CAFs promote cancer cell proliferation, migration, and invasion by actively oxidizing fatty acid synthase (FAs) via upregulation of CPT1A and engaging in minimal glycolysis [143]. Another study by Peng et al*.* found that elevated CAF expression enhances CRC cell membrane motility, which in turn increases glucose uptake and metabolism in CRC cells [144]. Recently, two CAF subtypes were identified in pancreatic tumors: inflammatory CAFs (iCAFs), which express nuclear factor-κB signaling and inflammatory cytokines/chemokines, and myofibroblastic CAFs (myCAFs), which express α-smooth muscle actin and matrix proteins [145, 146]. Similar CAF population heterogeneity has been observed in breast and lung cancers [147, 148]. Although the specific involvement of iCAFs, myCAFs, or other CAF subtypes in peritoneal metastasis has yet to be elucidated, the overall role of CAFs in this process is undeniably crucial.

In summary, effective communication and interactions among immune cells, the host microenvironment, and cancer cells play a crucial role in the intricate process of generating organ-specific metastatic sites. The dynamic interplay between these components orchestrates a complex series of events that determine the successful establishment and growth of metastases.

## Deciphering organ-specific metastasis in CRC: crucial molecules and pathways

**Fig. 5:**
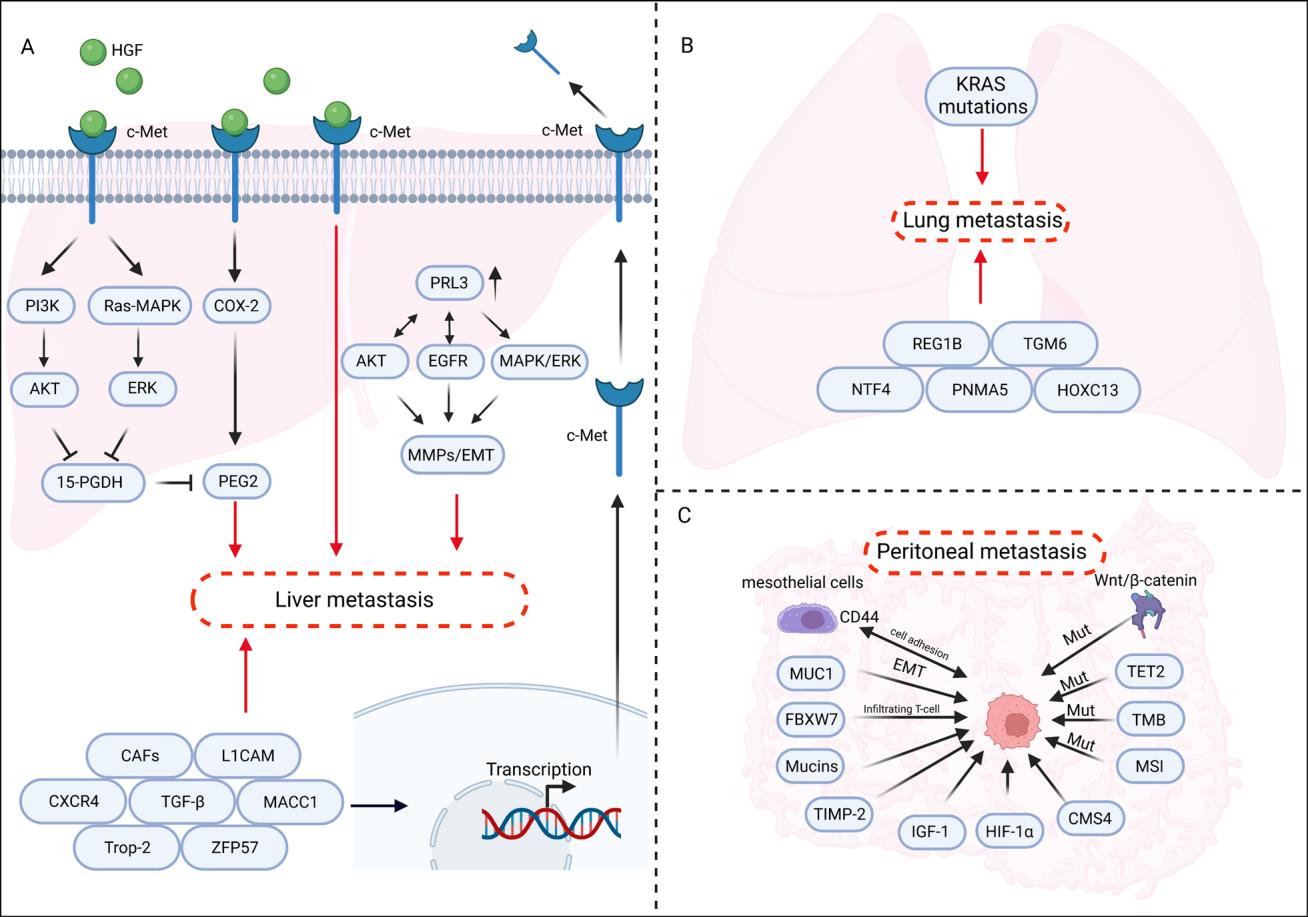
A schematic illustration of key molecular signaling pathways in metastasis. **A** By overstimulating the phosphatidylinositol-3-kinase (PI3K) and mitogen-activated protein kinase (MAPK) signaling pathways, the HGF/c-Met signaling pathway advances cancer cell metastasis and causes 15-PGDH downregulation. PRL3 transcripts are found in abundance in CRC liver metastases, and the PRL3-induced augmentation of EMT is contingent upon EGFR activation. Additionally, PRL3 enhances cell invasion and increases MMPS expression by activating AKT. In conjunction with these factors, L1CAM, TGF-β, MACC1, CXCR4, Trop-2, CAFs, and ZFP57 are also implicated in the progression of CRC liver metastasis. **B** Certain oncogenes are associated with lung metastasis in colorectal cancer (CRC). KRAS mutations often lead to lung metastasis, whereas its wild-type counterpart tends to result in liver metastasis. Genes REG1B, TGM6, NTF4, PNMA5, and HOXC13 have been identified as key candidates associated with colon cancer lung metastasis. **C** Certain molecular pathways are involved in colorectal cancer's peritoneal metastasis. A low mutation load in tumor suppressor gene FBXW7 is observed in this metastasis type, whereas a high mutation load of FBXW7 in primary tumors associates with less metastasis. Mucin glycoproteins, like CD44 and MUC1, play crucial roles in metastasis, with the latter promoting EMT and inhibiting cell apoptosis. Other proteins, including TIMP-2, IGF-1, and HIF-1α, increase in peritoneal metastasis, aiding tumor cell settlement in the subperitoneal region. An enrichment of CMS4 subtype is noted in primary tumors with peritoneal metastasis, while peritoneal metastasis presents comparable KRAS mutation rates to primary tumors but shows higher prevalence of GNAS and BRAF pathways. Transcriptome analysis reveals peritoneal metastasis has more mutations in the Wnt/β-catenin signaling pathway's negative regulators, TET2 mutations, mismatch repair gene mutations, and increased tumor mutational burden compared to primary tumors

CRLM could encompass numerous molecules and signaling cascades, such as the hepatocyte growth factor (HGF)/cMet signaling pathway, phosphatase of regenerating liver (PRL3), TGF signaling, L1 cell adhesion molecule (L1CAM), C-X-C chemokine receptor type 4 (CXCR4), Cancer-Associated Fibroblasts (CAFs), and tumor-related calcium signal transducer 2 (Trop-2) (Fig. 5A). The tyrosine kinase c-Met is substantially expressed in liver metastasis. Met, a receptor for HGF, correlates positively with the tumor stage of CRC hepatic metastases [149]. HGF/C-Met inhibition diminishes CRLM proliferation and invasion [150]. Additionally, HGF/C-Met orchestrates the COX-2/PGE2 pathway, elevating PGE2 synthesis via COX-2 overexpression and impeding PGE2 degradation through Ras-MAPK/ERK, whereas HGF-driven 15-PGDH downregulation is facilitated by PI3K/AKT signaling. CRC liver metastases have an abundance of PRL3 transcripts. However, initial tumors with no potential for metastasis and normal colonic epithelium cannot express PRL3 [151]. Activating AKT and EGFR enables PRL3 to drive cell invasion and upregulate MMPS and EMT. Another study by Al-Aidaroos and Lee et al*.* indicated that PRL-3 is related to PI3K/AKT and MAPK/ERK, providing a direct mechanism of liver metastasis. The PRL-3-induced liver metastasis was shown to be mediated by lymph node metastases and increased tumor markers (CEA and CA19-9) in the blood [152, 153]. PRL3 promotes the metastasis of cancer cells by modulating a variety of prometastatic effector molecules via AKT, EGFR, PI3K/AKT, and MAPK/ERK. More than 30 members of the TGF-β superfamily are involved in TGF-β signaling [154], with TGF-β being the most essential. Similarly, epidermal growth factor-like domain protein 6 (MEGF6) promotes EMT in CRC via transforming growth factor (TGF-β)/SMAD [155]. Additionally, TGF-β reduces E-cadherin expression and increases vimentin expression, leading to EMT that fosters CRC invasion and migration [156]. In addition, CRC-derived CXCR4 stimulates (hematopoietic stem cells) HSCs, producing SDF-1 and increasing CRC cell liver metastasis [157]. In addition to activating STAT3 via the production of IL-11 by CAFs, it can also induce EMT by influencing gene transcription b SMAD in CRC cells. And after epithelial integrity is compromised, the expression of L1CAM is elevated, leading to tumor development and metastasis, notably liver colonization [158, 159]. Trop-2 and ZFP57 expression are also required for the invasiveness of CRC cells, which has been found to facilitate CRC liver metastasis [160, 161].

Similar to the association of specific oncogenes with CRLM, certain oncogenes are also involved in the development of colorectal cancer lung metastasis. (Fig. 5B) Advances in next-generation DNA sequencing and transcriptome analysis have made precision medicine possible for treating lung metastasis in colorectal cancer. Moorcraft et al*.* wanted to molecularly characterize CRC lung metastases and determine whether their molecular profiles were concordant with the source tumor. It was found that APC (71%), KRAS (58%), and TP53 (46%) were the most commonly altered genes. In an alternative large-scale investigation, Kim and team examined KRAS mutations in primary tumors and associated metastases, identifying significant distinctions in initial metastatic patterns tied to KRAS mutational status. As a metastatic site, KRAS mutation tumors tended to develop lung metastases more frequently, whereas KRAS wild-type tumors tended to develop liver metastases more frequently [162]. A study by Cejas and colleagues found that lung metastatic patients had a greater percentage of KRAS mutations than patients with metastatic liver cancer (59% vs. 32%). Several studies have found that KRAS mutations reduce the time-to-lung metastasis [163, 164]. After comprehensively analyzing CRC-related data in the TCGA database, researchers identified a set of key candidate genes (REG1B, TGM6, NTF4, PNMA5, and HOXC13) associated with colon cancer lung metastasis [165].

Similarly, there are specific molecular pathways involved in the peritoneal metastasis cascade of colorectal cancer. (Fig. 5C) FBXW7 serves as a pivotal tumor suppressor gene, displaying a comparatively low mutation load in colorectal cancer accompanied by peritoneal metastasis (CRCPM). Intriguingly, in contrast to in situ colorectal tumors, an elevated mutation load of FBXW7 in primary colorectal cancer (CRC) has been corroborated to be linked with the lack of distant metastasis, augmented tumor-infiltrating T-cell proliferation, and bolstered antigen presentation [166–168]. An extensive array of preclinical investigations has uncovered the vital function of mucin glycoproteins in the peritoneal metastasis of colorectal cancer. For example, CD44 facilitates cell adhesion between CRC tumor cells and peritoneal mesothelial cells, with its heightened expression primarily restricted to late-stage CRC [169]. Similarly, MUC1 exhibits overexpression in colorectal cancer, promoting epithelial-mesenchymal transition (EMT) while suppressing cell apoptosis [170]. Other proteins, including TIMP-2, IGF-1, and HIF-1α, demonstrate increased expression in clones of peritoneal metastasis from colorectal cancer as opposed to primary colorectal tumors or liver metastases originating from colorectal cancer. These proteins play a role in fostering tumor cell settlement within the subperitoneal region [25, 171]. Through the collection of fresh frozen tissue specimens from the primary colon cancer lesion and matched peritoneal metastases of patients who underwent, the authors have identified the CMS4 subtype as participating in the progression of peritoneal metastasis in colorectal cancer. Pathological characteristics of the tissue were analyzed and the CMS4 status of all lesions was determined using reverse transcription quantitative PCR. The study by Ubink and colleagues revealed a significant enrichment of CMS4 in primary tumors with peritoneal metastasis, demonstrating significant heterogeneity in this patient subgroup [172, 173]. Additionally, an integrative analysis of tumors demonstrated that, in comparison to primary colorectal cancer (pCRC), peritoneal metastasis (PM) features a comparable KRAS mutation rate, but with an increased incidence of GNAS (mucinous) and BRAF (non-mucinous) pathways. No differences in microsatellite instability (MSI) or tumor mutational burden (TMB) were identified between PM and pCRC tumors. [167] Interestingly, another transcriptome analysis revealed unique characteristics of isolated metastatic peritoneal carcinoma in comparison to primary colorectal cancer. These features include a higher prevalence of mutations in negative regulators of the Wnt/β-catenin signaling pathway, TET2 mutations, mismatch repair gene mutations, and an increased tumor mutational burden [174].

In summary, the exploration of molecular mechanisms and gene alterations associated with colorectal cancer metastasizing to the liver, lungs, and peritoneum underscores their criticality in advancing targeted therapeutic strategies and propelling precision medicine.

## Targeting metastatic organotropism for CRC therapy

While therapeutic strategies for metastatic CRC (mCRC) have witnessed significant advancements over the past two decades, thereby improving the overall survival (OS) rate of patients, metastasis continues to be a leading cause of cancer-related mortality. The interplay between metabolic reprogramming, the function of tumor-derived exosomes, and the activation of multiple immune system components, all of which are intricately tied to metastasis, could potentially enhance these treatment methods [175]. Gaining a comprehensive understanding of these interactions paves the way for the development of more refined treatment strategies, which could potentially lead to a further improvement in patient prognosis.

### Targeting metabolic adaptation

Due to the importance of metabolic reprogramming in tumor metastasis, tumor cells are equipped to use metabolites from the microenvironment to their advantage, allowing them to adapt to the unfavorable conditions encountered during the metastatic cascade. Consequently, targeting the metabolism of metastases may be an effective technique for treating metastatic cancer.

### Targeting glycolysis

Utilizing the “Warburg effect,” tumor cells can rapidly fluctuate their energy requirement and adapt to their microenvironment. Inhibition of glycolysis may prevent metastasis of cancer cells. For instance, as a glycolysis inhibitor, 2-deoxyglucose (2-DG) prevents the conversion of glucose to glucose-6-phosphate by competitive inhibition. The administration of 2-DG to CRC cells lowered their 5-fluorouracil resistance, resulting in a decline in glycolysis-related enzyme expression and cell invasiveness, as well as the inhibition of EMT-associated cytokine secretion and inactivation of integrin and metalloproteinases 10 and 17 [176]. Multiple studies have shown that the glycolysis inhibitor (LND) can make CRC cells more sensitive to chemotherapy treatments. LND suppressed brain cancer metastasis in mice models of lung cancer without causing toxicity, even during long-time feeding [177]. In addition, in vitro and in vivo analyses, a mitochondria-targeted LND inhibited bioenergetics, ROS generation, and AKT/mTOR/p70S6K signaling from limiting metastasis. The application of 2-DG or 3-BrPA impeded glycolysis and breast cancer cell metastasis by diminishing vimentin, Snail, Slug, and Twist, while enhancing E-cadherin expression, thereby reversing the EMT induced by NQO1 [178].

### Targeting amino acid metabolism

In the progression and metastasis of CRC, amino acid metabolism plays a crucial role by providing carbon for the tricarboxylic acid cycle, nitrogen for base synthesis, and maintaining redox equilibrium. This metabolic process involves various amino acids such as glutamine, glycine, and tryptophan, etc. Just like glucose, amino acids play a crucial role in energy generation and biosynthesis within tumor cells. Therefore, targeting amino acid metabolism has become a significant area of focus in contemporary oncology research and treatment [179].

Cells with the PIK3CAp^110α^ mutation are more sensitive to the glutamine in their environment, indicating a potential vulnerability in CRC [180]. Preclinical and clinical trials have demonstrated that combined treatment with GLS inhibitor CB-839 and capecitabine significantly improves therapeutic efficacy against CRC cells harboring the PIK3CAp^110α^ mutation [181]. In addition, the investigation of glutamine metabolism as a therapeutic approach for KRAS-mutant colorectal cancer (CRC) has advanced significantly. In conditions of glutamine scarcity, KRAS-mutant CRC cells demonstrate adaptability by escalating the activity of asparagine synthetase and increasing asparagine production. Research findings indicate that the combined application of L-asparaginase and rapamycin markedly inhibits the proliferation of KRAS-mutant tumors in vivo. It has been reported that vitamin C can stimulate the depletion of glutathione, leading to an oxidative stress response that counteracts the growth and metastasis of KRAS and BRAF-mutant CRC under conditions of high glycolysis [182]. The inhibitors targeting SHMT1/2 aim to disrupt glycine metabolism. SHIN1 inhibits glycine synthesis, leading to a decrease in purines and triphosphonucleosides, which further hinders cell growth [183]. The enzyme MTHFD2 enhances the aggressive characteristics of CRC cells. Its counteracting agent, LY345899, has demonstrated promising therapeutic attributes and is therefore considered a viable candidate for future clinical studies [184]. As a standard therapeutic strategy, tryptophan metabolic enzymes serve as widely adopted treatment targets, offering substantial promise in the realm of tumor therapy. Despite this, the results of clinical trials relying on these enzymes have frustratingly shown inconsistency [185]. 1-L-MT (1-L-Methyltryptophan), a competitive inhibitor targeting IDO1, has the potential to suppress the proliferation of human colorectal cancer cells by inducing mitotic cell death [186]. The IDO1 inhibitor epacadostat is extensively employed in the clinical investigation of numerous types of tumors, including colorectal cancer [187]. Clinical trials, either completed or underway, have assessed the combinatory efficacy of ipatasertib and other anticancer drugs; these combination therapies include the pairing of ipatasertib with parbociclib and azacitidine (NCT03182894), or in tandem with MK-3475 (NCT02178722). A dual inhibitor targeting IDO1/TDO, HTI-1090, has been introduced into clinical trials for late-stage solid tumors, including colorectal cancer (NCT03208959). AhR, a transcription factor exhibiting activity in the cytoplasm, plays a pivotal role in regulating immune responses and cellular differentiation. Recent studies have revealed that AhR can mediate multiple crucial functions through binding with certain tryptophan intermediates [185]. As reported in research, the AhR antagonists BAY2416964 and IK-175 can specifically bind with AhR in late-stage solid tumors, subsequently inhibiting its activation (NCT04069026 and NCT04200963) [188].

### Targeting lipid metabolism

There is evidence that abnormal lipid metabolism plays a critical role in the development of colorectal metastases across a broad spectrum [189]. Elevated levels of lipogenic enzymes are often observed in people with advanced metastatic CRC, suggesting that targeting these enzymes through lipid metabolism-centric therapies could offer a distinct treatment approach for CRC [190].

FASN overexpression is associated with worse clinical outcomes in CRC patients, which involves producing long-chain fatty acids from scratch [191, 192]. Cerulenin, identified as the initial FASN inhibitor, was originally employed as an antifungal antibiotic, showcasing its versatility in medical applications [193]. Cerulenin was discovered to not only suppress liver metastasis of CRC cells in mice but also to enhance the therapeutic impact of oxaliplatin, a third-generation platinum compound, on CRC [194]. In addition to cerulenin, luteolin (3,4,5,7-tetrahydroxyflavone), TVB-3664, and epigallocatechin-3-gallate (EGCG) are all potential FASN inhibitors found in medicinal herbs, considered to exercise its anticancer effect in CRC via modulating multiple tumor signaling pathways, including IGF-1, AKT, STAT, Erk1/2, and Wnt-β-catenin [195–198]. Specifically, by augmenting miR-384 and decreasing PTN levels, luteolin curtailed the migration and invasion of CRC cells in vitro and in vivo, underscoring its prospective therapeutic relevance [197]. TVB-3664 hindered tumor formation in CRC cells and the patient-derived xenograft (PDX) model by manipulating the AKT and Erk1/2 cancer-promoting pathways, leading to the altered lipid composition of tumors and indicating its possible therapeutic significance [196]. The findings of Luo's investigation showed that EGCG curtailed CRC cell invasion through the inhibition of STAT transcription factors, highlighting its prospective significance in cancer treatment strategies. Additionally, this chemical may be delivered orally or externally without creating side effects, and it may be employed as a safer alternative to existing anticancer medications.

Besides FASN, a variety of enzymes participating in the de novo production of fatty acids exhibit tumor-enhancing properties in CRC, rendering them appealing targets for therapy. Employing a small-molecule inhibitor to target ATP citrate lyase (ACLY) and CD36, a fatty acid transporter (also referred to as fatty acid translocase), has demonstrated therapeutic benefits [57, 199]. Cholesterol belongs to the sterol family. A meta-analysis suggested that reducing cholesterol metabolism using statins, which are HMG-CoA reductase inhibitors, lowered overall and cancer-specific mortality rates in CRC patients [200].

### Targeting exosomes

As exosomes play a significant role in how cancer spreads and are easily obtained during liquid biopsies, they can be used in a number of therapeutical applications. Since circulating exosomes reflect the condition of tumor cells, their features may provide crucial information for prognosis and therapy decisions. For instance, exosomes, glypican-1 (GPC1), are sensitive indicators for identifying early pancreatic cancer lesions [201]. In addition, exosomes containing immune checkpoint proteins such as PD-1 from T and dendritic cells can indicate a good response to immune checkpoint treatment in metastatic melanoma patients, allowing for early cancer detection, diagnosis, and treatment [202].

Exosomes may be attractive therapeutic targets for malignancies with metastatic disease. For instance, a screen of molecules targeting exosome formation discovered numerous pharmacological agents that inhibited tumor-derived EVs, indicating that these medications might be repurposed for metastatic anticancer treatment [203]. A number of SRC-like inhibitors are currently used in the clinics, such as dasatinib or bosutinib, as SRC is an important promoter of cancer exosomes. It would be essential to evaluate the role of exosome inhibition to determine the anticancer effects of these agents [204]. Finally, by interfering with the establishment of the tumor microenvironment or PMN, drugs that inhibit tumor exosome uptake may also have therapeutic utility. For instance, exosomes from melanoma cells suppress type I IFN receptors and induce the production of IFN-inducible cholesterol 25-hydroxylase (CH25H) in recipient cells. Importantly, CH25H inhibition led to increased exosome absorption by resident cells and the establishment of a PMN [205]. These various prognostic and therapeutic techniques involving exosomes are potential cancer treatments.

### Targeting immune cells

As a result of progress in immune checkpoint investigations in numerous cancers, immunotherapy has gained considerable interest as a potential treatment method for CRC cases characterized by DNA mismatch repair deficiencies (dMMR)/high microsatellite instability (MSI-H) and heightened tumor-infiltrating lymphocyte (TIL) presence. However, for CRC patients with high mismatch repair capability (pMMR) and low microsatellite instability (MSI-L), immune checkpoint treatment has not shown significant benefits [206]. In fact, immunotherapy is now commonly used in oncology, improving cancer prognosis [207]. As an illustration, PD-1, PD-L1, and CTLA-4 act as negative modulators of T cells, serving an essential purpose in controlling immune reactions and averting autoimmunity through the dampening of T cell activation [208]. For instance, in a Phase II study conducted in 2015, pembrolizumab, an anti-PD-1 inhibitor, demonstrated significant efficacy across all patients with mismatch repair deficiencies (dMMR), including those with metastatic colorectal cancer (mCRC) [209]. This was further confirmed in a subsequent Phase III clinical trial, where pembrolizumab not only outperformed conventional chemotherapy in patients with MSI-high dMMR mCRC, but also provided long-term relief for dMMR CRC patients even in frontline treatments [210]. Ultimately, compared to chemotherapy, monotherapy with pembrolizumab has shown clinically meaningful improvements in patients' quality of life [211]. In a noteworthy phase II study, a PD-1 inhibitor, dostarlimab, showed a complete response rate of 100% among twelve patients with late-stage dMMR rectal cancer. After six months of treatment, all patients demonstrated full clinical and pathological remission, with no evidence of residual tumor found in colonoscopies and tissue biopsies [212]. Currently, additional research is underway to assess the effectiveness of combining immunotherapies with chemotherapy, targeted therapy, or other immunotherapeutic drugs. The efficacy of combination therapies in improving survival rates in patients with MSI-high dMMR mCRC and untreated mCRC has been confirmed in two phase II trials, CheckMate 142 (ipilimumab, a CTLA-4 inhibitor) and AtezoTRIBE (ipilimumab, a CTLA-4 inhibitor) [213, 214]. Furthermore, another phase II trial demonstrated the improvement in remission and overall survival periods when the PD-1 antibody, tislelizumab, was used in combination with the multi-kinase inhibitor, regorafenib [215]. In addition, Jiang et al.'s clinical research demonstrated that PD-L1 may potentially synergistically guide immunotherapy for CRLM, potentially resulting in significant therapeutic benefits for patients suffering from CRLM [216]. Similarly, another study by Leach et al*.* revealed that blocking CTLA-4 with an anti-CTLA-4 monoclonal antibody boosted the anticancer immune response [217]. Hence, immunotherapy may provide patients with colorectal cancer additional treatment options.

## Conclusion

Great efforts have been made to understand the molecular mechanisms and genetic events driving disease progression. Particularly within the "seed and soil’’ hypothesis framework, a growing number of molecular and cellular mechanisms for CRC metastasis organotropism have been uncovered. The uniqueness of the metastatic process in each organ is mainly based on metabolic reprogramming, exosomes, and the immune microenvironment. Despite advances in the detection and diagnosis of CRC using a combination of clinical profiling data and experimental models, the present studies are unable to respond appropriately and constantly to this complex metastatic process. Therefore, our understanding of metastasis remains limited. The molecular mechanisms of organotropism still have to be discovered.

As a whole, metastatic organotropism is a complex process that requires more than a few months to decades and results from interactions between numerous factors. Consequently, targeting multiple pathways at once and employing the combination therapy strategy could be vital in combating cancer. Individualized therapies could be designed to block or slow the progression of metastases in various organs. A large number of innovative techniques, including targeted sequencing, SNP arrays, ctDNA sequencing, whole-exome sequencing, RNA sequencing, and gene expression analysis, are contributing to the diagnosis and treatment of CRC metastases. Further extensive studies and implementation of these new technologies may provide future directions in treating metastatic CRC.

## Data Availability

As this is a review article, it does not contain any new data from the authors' experiments. All data and materials referenced in the article are appropriately cited.
